# Local Cell Death Changes the Orientation of Cell Division in the Developing *Drosophila* Wing Imaginal Disc Without Using Fat or Dachsous as Orienting Signals

**DOI:** 10.1371/journal.pone.0167637

**Published:** 2016-12-28

**Authors:** Abhijit Kale, Gerard Rimesso, Nicholas E. Baker

**Affiliations:** 1 Department of Genetics, Albert Einstein College of Medicine, 1300 Morris Park Avenue, Bronx, NYC, NY, United States of America; 2 Department of Ophthalmology and Visual Sciences, Albert Einstein College of Medicine, 1300 Morris Park Avenue, Bronx, NYC, NY, United States of America; 3 Department of Developmental and Molecular Biology, Albert Einstein College of Medicine, 1300 Morris Park Avenue, Bronx, NYC, NY, United States of America; University of Dayton, UNITED STATES

## Abstract

*Drosophila* imaginal disc cells exhibit preferred cell division orientations according to location within the disc. These orientations are altered if cell death occurs within the epithelium, such as is caused by cell competition or by genotypes affecting cell survival. Both normal cell division orientations, and their orientations after cell death, depend on the Fat-Dachsous pathway of planar cell polarity (PCP). The hypothesis that cell death initiates a planar polarity signal was investigated. When clones homozygous for the *pineapple eye* (*pie*) mutation were made to initiate cell death, neither Dachsous nor Fat was required in *pie* cells for the re-orientation of nearby cells, indicating a distinct signal for this PCP pathway. Dpp and Wg were also not needed for *pie* clones to re-orient cell division. Cell shapes were evaluated in wild type and mosaic wing discs to assess mechanical consequences of cell loss. Although proximal wing disc cells and cells close to the dorso-ventral boundary were elongated in their preferred cell division axes in wild type discs, cell shapes in much of the wing pouch were symmetrical on average and did not predict their preferred division axis. Cells in *pie* mutant clones were slightly larger than their normal counterparts, consistent with mechanical stretching following cell loss, but no bias in cell shape was detected in the surrounding cells. These findings indicate that an unidentified signal influences PCP-dependent cell division orientation in imaginal discs.

## Introduction

Oriented cell division influences how animal tissues grow, especially in tissues where cells are not very motile[1–4]. It is also hypothesized that the orientation of cell division can release mechanical tensions that arise during growth[5–7].

Previously, we reported that the orientation of division in wing imaginal discs from *Drosophila* is altered in the vicinity of apoptotic cells[8]. Mitotic spindles tend to become re-oriented towards locations where cell death occurs, leading to a division axis towards the dying region (division axis refers to the direction in which the daughter cells separate whereas the division plane, where the new cell boundary forms, is at right angles to the division axis). This effect of cell death required the Fat-Dachsous planar polarity system[8]. Fat and Dachsous are also required for the normal patterns of division orientation in wild type wing discs, which suggests that a common mechanism may control the orientation of normal divisions and their reorientation in response to cell death [4, 8]. The source of the spatial information that orients cell divisions in normal wing development is not yet clear, although there may be roles for mechanical forces and junctions with neighboring cells [9, 10]. By contrast, the dying cell is presumed to be the direct or indirect source of the hypothesized signal that orients cell division in response to local cell death, providing a system to investigate the source of orienting signals.

Fat and Dachsous are large proto-cadherin molecules in the plasma membrane that can mediate heterophilic cell adhesion. They are required for the planar cell polarity of differentiated epithelial cells that is revealed through the positioning of certain sub-cellular structures, such as the wing hair structures that are produced at the apical surface of each cell in the wing blade [11, 12]. Mutations in both *fat* and *dachsous* also enhance growth, through effects on the Salvador-Hippo-Warts pathway of tumor suppressors [13–15]. Effects of Fat and Dachsous on planar cell polarity are mediated by the atypical myosin Dachs and by Atrophin and Fbxl7. The latter two proteins bind to the intracellular domain of Fat [16, 17]. Dachs and Fbxl7 also affect growth, whereas cells lacking Atrophin grow similarly to controls, but lack proper cell division orientation [8, 17–19]. Fat affects growth cell-autonomously and behaves as a receptor for Dachsous [20]. However, there are also circumstances where Dachsous appears to respond as a reciprocal receptor for Fat[21]. Dachsous is expressed in gradients in imaginal discs that are thought to define PCP, in conjunction with a reciprocal gradient of Four Jointed, a Golgi protein that phosphorylates the Fat and Dachsous extracellular domains[22–24].

**Fig 1:**
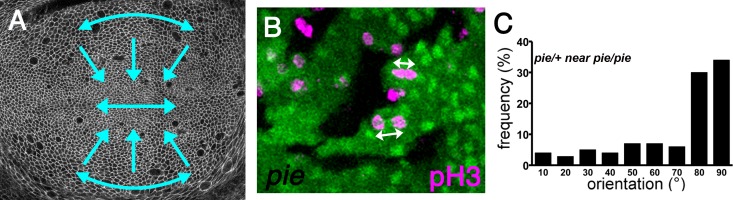
Cell divisions in the wing disc. **A,** Wing pouch region of the wing disc. Cells outlined with anti-Dlg. Typical orientations of cell divisions are indicated by blue arrows, as described in previous studies. In the center of the disc, cells tend to divide parallel to the dorso-ventral boundary. Most cells divisions in the wing disc point distally, towards the center of the DV boundary. Peripheral cells in the proximal wing pouch tend to divide circumferentially[4]. **B**, Enlarged portion up of a wing disc contained cell clones homozygous for the *pie*^*E1-16*^ mutation (lacking GFP label). Mitotic figures labeled with anti-pH3 (magenta). Selected mitotic division orientations indicated by arrows (white). **C**, When the orientation of nearby cell divisions is measured with respect to clones homozygous for the *pie*^*E1-16*^ mutation, in which chronic cell death occurs[38] there is a strong bias for cells to divide towards nearby clones [8]. By contrast an almost random distribution is seen near wild type clones because even though division may be locally biased, clone boundaries vary so that the two bear little relationship [8]. Examples of the cell division orientation distributiion near clones that do not re-orient divisions are shown in Fig 3A–3C of this paper.

Fat, Dachsous and Atrophin are required for division orientation in the normal developing wing[4, 8]. During the third instar larval stage, cells dividing in the wing pouch region of the wing disc tend to divide along the proximo-distal axis, and this contributes to the proximodistal elongation of clones of cells growing during this period (Fig 1A)[4]. There are other preferred orientations of cell division elsewhere and at other stages. For example, cells at the periphery of the wing pouch, which will contribute to the proximal wing and the wing hinge, tend to divide circumferentially, i.e. orthogonal to the proximo-distal axis. In addition, cells adjacent to the dorso-ventral wing boundary that runs across the wing pouch tend to divide parallel to this boundary, in sharp distinction to other cells of the wing pouch(Fig 1A)[4]. All these divisions become more random in orientation without Fat, Dachsous, or Atrophin, and clones of cells mutant for these genes are more rounded than elongated[4, 8].

The relationship of cell division orientation to cell death first came to attention in studies of cell competition. Cell competition refers to loss of cells from genetic mosaics that would survive in a homogenous environment, such as the loss of cells heterozygous for ribosomal protein mutations (‘Minute’ cells), or of cells near to cells with higher copy numbers of the *myc* gene[25–28]. The disadvantaged cells often undergo apoptosis in the competitive environment[26, 27, 29]. Both competitive situations are associated with progressive intermingling of the competing cell populations to a greater degree than is observed between non-competing cell populations [8, 30, 31]. In the case of competition between wild type and Minute cells, competing wild type cells tended to re-orient their cell division axes perpendicular to the boundary with Minute cells, with one daughter cell closer and one more distant [8]. When baculovirus p35 was expressed to prevent cell death, both the division orientation and the intermingling were reduced, suggesting that cell death re-orients nearby cell divisions through the Fat-Dachsous pathway and potentially affects the shapes of descendent cell clones. Consistent with this hypothesis, cell divisions were also re-oriented near clones of cells homozygous for a mutation, *pie*, that leads to a chronically elevated cell death rate [8]. Surprisingly, however, enhanced intermingling of competing cell populations with different *myc* levels was recently observed in the pupal notum, at a stage where very little cell division occurs[31].

Several studies indicate that mechanical forces may affect cell division orientation. It has long been known that the division plane tends to bisect the long axis of the cell [32, 33]. Cell shape is affected by the forces acting upon cells, which include forces exerted by neighboring cells. Within the wing disc, cells in the wing pouch region lack a consistent shape but cells at the periphery of the wing disc that will give rise to the proximal wing and the wing hinge are stretched in a circumferential manner that correlates with their cell division orientation [4, 34]. This stretching is thought to be due to the growth of the central wing pouch region during the larval growth phase. Because the periphery of the disc does not grow fast enough to accommodate the central expansion, cells of the central wing pouch are relatively compressed whereas those of the periphery are stretched[10, 35, 36].

In this study we manipulated the genotypes of dying cell populations in mosaic clones in order to test for possible instructive roles of the Fat/Dachsous planar cell polarity pathway and morphogen signaling pathways in orienting cell division. We found no evidence for instructive roles of known signaling molecules on the changes in cell division orientation that accompany local wing disc cell death and instead discuss the possibility that mechanical consequences of local cell loss might affect cell division orientation.

## Materials and Methods

### Mutant and transgenic strains were used as follows

*pie*^*E1-16*^ was obtained from Dr. T. Grigliatti and characterized molecularly in our laboratory[37, 38]; *ds*^*UA071*^[39] was obtained from the Bloomington Drosophila Stock Center; *ft*^*NY1*^ was isolated in our laboratory[40]; *dpp*^*d12*^ and *wg*^*RF*^ were obtained from Dr. G. Morata[41]; *P{arm-LacZ}* transgenes were obtained from BDSC[42]. Mitotic clones were induced using heat shock induction of Flp recombinase[43]. Recombination was induced 60±12h after egg laying and larvae dissected for clone examination 60h later.

Antibodies included mAb40-1a (ßgal) and mAb4F3 (Dlg) from Developmental Studies Hybridoma Bank and rabbit anti-pH3 form Cell Signaling Technology. Immunochemistry procedures have been described[44]. Orientation of cell divisions was measured as described previously[8]. Mitotic figures were visualized by anti-pH3 labeling of wing imaginal discs and the orientation of anaphase and telophase figures recorded. The orientation with respect to nearby clone boundaries was assessed as the angle between the direction of chromosome segregation (the division axis) and the nearest clone boundary, defined as the line connecting the midpoint of the boundary faces of the two flanking cells[8].

Cell shapes were recorded by confocal microscopy of wing imaginal discs labeled for anti-Dlg. Where junctional Dlg protein was expressed in different z planes across the disc, appropriate z planes were focus-stacked using Adobe Photoshop CS5. Images were made binary using thresholds in Image J (v1.44j) and the angle of the cells long axes recorded using the fit ellipse measurement. Since this software records an angle of 0° for perfectly round cells, such cells were excluded by requiring a long axis: short axis ratio >1.25.

To measure apical cell size, mutant clones or wild type territories were traced, areas measured using Image J (v1.44j) and divided by the number of cells.

To assess the orientation of cells surrounding *pie* homozygous clones, cells adjacent to *pie* clones were outlined by anti-Dlg labeling and the longest cell axis determined. The angle made with the clone boundary was determined using the same method described for cell division orientation. Control cells were selected from cells at the same proximo-distal position in the disc.

## Results

A previous study described oriented cell division in response to cell death that depends on the planar cell polarity genes *ft*, *ds*, and *atro*[8]. This was first observed when death was due to cell competition of ‘Minute’ cells, heterozygous for a ribosomal protein mutation, at interfaces with wild type cell populations. The same phenomenon was seen when cell death was a cell autonomous consequence of loss of a PHD finger protein encoded by the *pineapple eye* gene(Fig 1B and 1C). Although the function of the *pie* gene is not known in detail, when the gene is mutated in imaginal disc clones then a high frequency of apoptosis results[38]. This provides a useful assay for signals produced by dying cells, since the rate of apoptosis is not sufficient to eliminate the *pie* mutant clones completely, which provide a continuous source of dying cells. The *pie* gene is also required for germline stem cell self-renewal, where it may affect BMP signaling[45].

**Fig 2:**
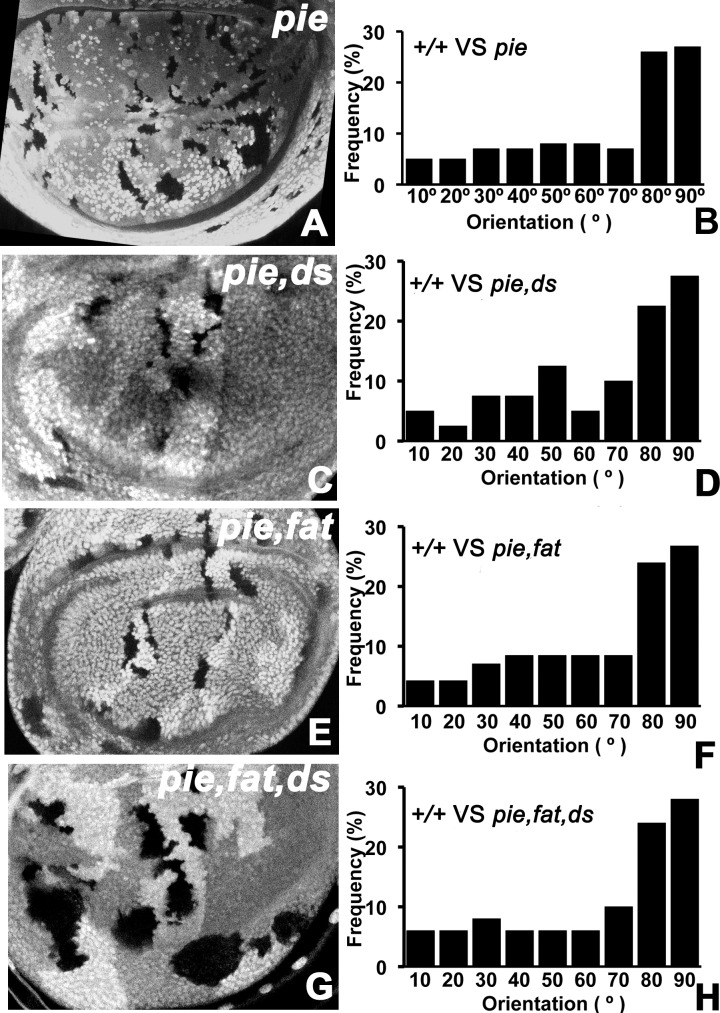
Cell division re-orientation is independent of Dachsous or Fat in the dying cells. Wing discs containing clones homoygous for *pie*^*E1-16*^ (A), *ds*^*UA071*^ *pie*^*E1-16*^ (C), *ft*^*NY1*^ *pie*^*E1-16*^ (E), and *ds*^*UA071*^ *ft*^*NY1*^ *pie*^*E1-16*^ (G). Mutant clones lack LacZ labeling. Genotypes: (A) *hsF*; *pie*^*E1-16*^ *FRT40A* / P{armLacZ} FRT40A; (C) *hsF*; *ds*^*UA071*^ *pie*^*E1-16*^ *FRT40A* / P{armLacZ} FRT40A; (E) *hsF*; *ft*^*NY1*^*pie*^*E1-16*^ *FRT40A* / P{armLacZ} FRT40A; (G) *hsF*; *ds*^*UA071*^ *ft*^*NY1*^ *pie*^*E1-16*^ *FRT40A* / P{armLacZ} FRT40A. B,D,F,H show the frequency of adjacent cell divisions with different orientations with respect to the clones. Clones of all these genotypes tend to orient nearby cell divisions.

In some assays, Fat behaves as a cell-autonomous receptor for Dachsous. It was therefore possible that Dachsous might be the activity coming from dying cells that orients nearby cell division. To test this model, clones of cells simultaneously mutant for both *pie* and *dachsous* were induced in wing discs. As has been described before, the majority of mitotic figures observed close to clones of *pie* mutant cells were oriented towards the clones (Fig 2A and 2B). By contrast, mitotic figures show little referred orientation near to wild type clones (although mitosis is oriented with respect to global axes in particular disc regions, clone boundaries take a variety of courses so show little correlation with mitotic axis[8]). When the dying cells lacked Dachsous expression, the orientation of division around *ds pie* clones was indistinguishable from that near *pie* clones, indicating that whatever polarizing signal resulted from cell death did not depend on the *ds* locus within the dying cell population (Fig 2C and 2D). We used the mutation *ds*^*UA071*^, a chemically-induced mutations that has been genetically characterized as a loss-of-function allele, [8, 39]. Although the *ds pie* mutant clones are smaller than *ds* mutant clones would be, presumably because of chronic cell death that occurs in *pie* mutants, mitotic clones mutant for *ds* induced at the same developmental stage clearly lost *ds* function, suggesting that Dachsous protein does not perdure for 60h following recombination (see ref [8]).

Although Fat is generally considered a receptor for Dachsous, there are also assays in which Dachsous appears to respond to Fat from neighboring cells[21]. To test whether *ft* might encode an orienting signal from dying cells, divisions were examined next to *ft pie* mutant clones. As was the case with *ds*, the clones of *ft pie* mutant cells continued to orient nearby cell divisions in a manner indistinguishable from clones mutant for *pie* alone (Fig 2E and 2F). We used the mutation *ft*^*NY1*^, a chemically-induced mutations that has been genetically characterized as a loss-of-function allele[8, 40].

Since both *ft* and *ds* can have signaling properties in certain assays, we considered the possibility that they might encode redundant signals from dying cells, and tested this model using *ds ft pie* triple-mutant clones. However, nearby wild type cells were oriented by *ds ft pie* mutant clones to the same degree as by *pie* clones (Fig 2G and 2H), as had been seen for each mutant individually. Therefore it does not seem that *ds* or *ft*, either individually or together, encode signals from dying cells that orient the division of other cells nearby. It was interesting that *ds ft pie* clones were larger than either *ds pie* or *ft pie* clones, suggesting greater hyperplasia in the absence of both *ft* and *ds* (Fig 2G).

Although these results argued against positive signaling roles for *ft* and *ds*, they did not rule out inhibitory signaling mechanisms. Thus one could envisage that, if apoptotic cells downregulated *ft* or *ds* expression before dying, then it could be this reduction in either or both protein that provided an orienting signal. If this were the case, then mutating *ft* or *ds* in dying cells would not affect orientation, because the mutation would mimic the effect of apoptosis in downregulating Ft or Ds expression.

**Fig 3:**
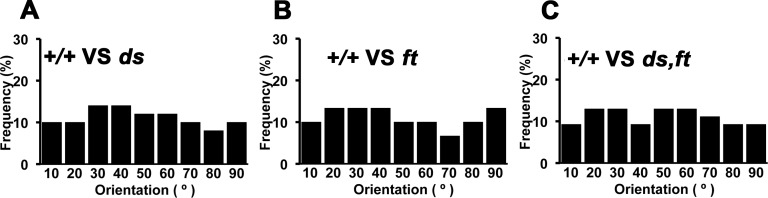
Loss of Dachsous or Fat is not sufficient to orient cell division. **A,** Frequency of adjacent cell divisions with different orientations with respect to clones homozygous for *ds*^*UA071*^, induced in the genotype *hsF*; *ds*^*UA071*^ *FRT40A* / P{armLacZ} FRT40A. **B** Frequency of adjacent cell divisions with different orientations with respect to clones homozygous for *ft*^*NY1*^, induced in the genotype *hsF*; *ft*^*NY1*^ *FRT40A* / P{armLacZ} FRT40A. **C** Frequency of adjacent cell divisions with different orientations with respect to clones homozygous for *ds*^*UA071*^ *ft*^*NY1*^, induced in the genotype *hsF*; *ds*^*UA071*^ *ft*^*NY1*^ *FRT40A* / P{armLacZ} FRT40A.

If it was reduced expression of *ds* or *ft*, alone or together, that provided the orienting signal from dying cells, then we would expect that clones lacking these genes would orient the division of nearby cells, mimicking the effects of cell death. To test this model, the mitotic orientation of wild type cells was measured near clones of *ds*, *ft*, or *ds ft* mutant clones. In all three cases the orientation of wild type cells was unaffected by proximity to the mutants, just as when wild type cells are near to wild type clones, and quite unlike wild type cells near to *pie* mutant clones (Fig 3). These results did not support the model that loss of Ft or Ds or both from apoptotic cells was a signal that orients the division of nearby cells.

**Fig 4:**
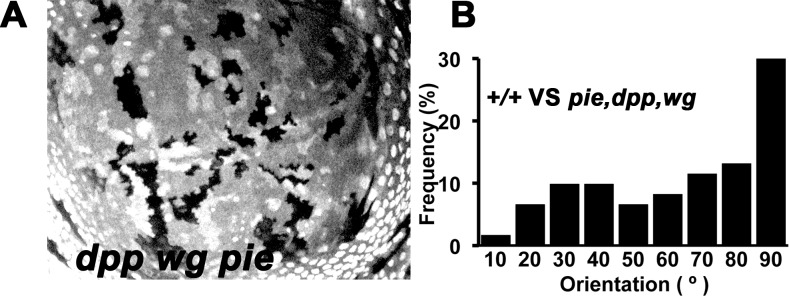
Cell division is not oriented by *dpp* or *wg*. **A,** Wing pouch containing clones homoygous for mutations in *dpp*, *wg* and *pie*. Mutant clones lack LacZ labeling. Genotype: *hsF; dpp*^*d12*^ *wg*^*RF*^ *pie*^*E1-16*^ FRT40A / P{armLacZ} FRT40A. **B**, Cell divisions near *dpp wg pie* homozygous clones frequently orient towards them, similar to cell divisions near *pie* homozygous clones (compare Fig 2B).

Since the data rule out contribution of Ds or Ft as signals in any simple way, cell-autonomous roles of *ds* and *ft* in the oriented cells may reflect responses to some other signal from dying cells. Two candidates are the secreted signaling molecules Decapentaplegic (Dpp) and Wingless (Wg)[46, 47]. Gradients of Dpp and Wg are responsible for multiple aspects of wing patterning, and previous studies have suggested they are both released by apoptotic cells and may contribute to proliferative responses to cell death[41, 48, 49]. Levels of Wnt signaling can influence cell division axis and planar cell polarity through the other PCP pathway dependent on Frizzled proteins [50]. To test whether Dpp and Wg released from dying cells orient the division of nearby cells, mitotic figures were examined in the vicinity of *dpp wg pie* triple mutant clones. Since both *dpp* and *wg* mutations act non-autonomously, mutant clones rarely affect patterning of the wing [51, 52]. The division of nearby cells continued to be oriented, as for *pie* clones, suggesting that Dpp and Wg were not required for the hypothesized orienting signal (Fig 4).

It cannot be ruled out that dying cells produce other biochemical signals that orient nearby cells. Having excluded the known signals, however, we also considered other mechanisms. It has long been known that cells tend to divide in the direction of their long axis, and this is also true for some cells in the wing disc[32]. If local apoptosis changes the shape of nearby cells, for example because nearby cells are stretched when apoptotic cells shrink and are removed, such cell shape changes might alter the orientation of cell division. In this kind of model, the effect of apoptosis on cell division would be indirect and due to the mechanical and morphological consequences of cell loss.

**Fig 5:**
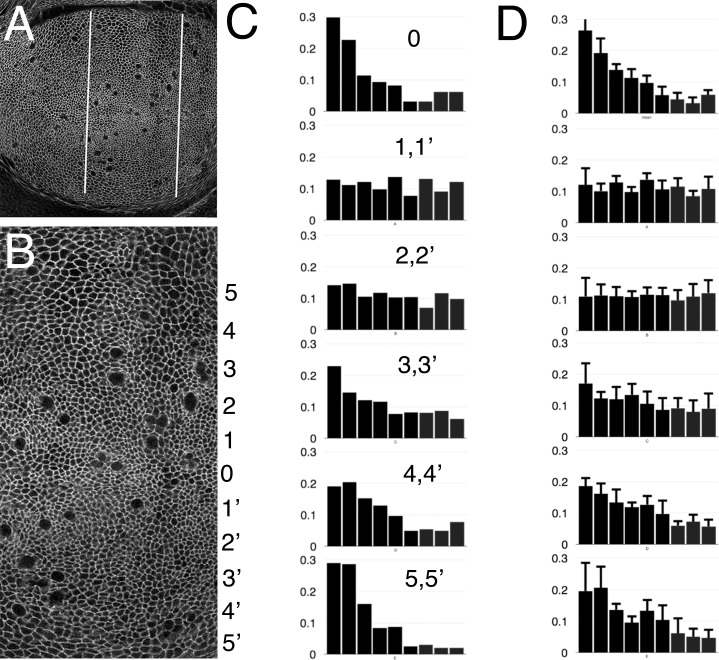
Cell shapes in the wing disc. **A**, Wing pouch region of the wing disc. Cells outlined by staining for Dlg protein. Mitotic cells occupy large apical profiles. Bars indicate the medial region enlarged in panel B. **B,** Cell shapes in the medial wing pouch. The long axis of cells was determined as a function of their distance from the dorso-ventral compartment boundary. This distance was recorded by dividing the dorsal and ventral medial regions of the wing discs into the dorso-ventral compartment region (zone 0) and five equally-wide zones arranged from distal to proximal (zones 1–5 dorsally and 1’-5’ ventrally). The larger area of proximal cells compared to distal is evident in this picture. **C**, Orientation of the cellular long axis and the dorsoventral compartment boundary. Cells were group in bins of 10°, where 0° represents cells elongated parallel to the dorsoventral boundary, 90° represents cells elongated perpendicular to it. The y-axis represents the proportion of cells exhibiting that orientation. Cells around the dorsoventral boundary (zone 0), as well as proximal cells (zones 4–5), both tend to parallel the dorsoventral boundary. There is no location where cells are predominantly oriented perpendicular to the boundary, although this is their preferred division orientation. Data for equivalent zones in the dorsal and ventral wing pouch were combined (eg 5 and 5’). **D**, Data averaged over 4 wing discs. Error bars represent 1 SEM.

To examine whether cell division orientation correlated with cell shape in normal development, we examined the shape of epithelial cells in the wing pouch region of the wild type, where most divisions are normally oriented towards the dorsoventral boundary that defines the future distal tip of the wing. Apical cell boundaries were revealed by labeling with an antibody against Discs Large, a membrane protein that localizes to septate junctions (Fig 5A and 5B)[53]. The direction of the long axis of each cell was identified (see Methods). When the apical orientation was plotted against the direction of the dorsal-ventral axis, preferred orientations were seen in some parts of the disc (Fig 5C and 5D). Specifically, cells near the dorso-ventral boundary were more often oriented parallel to the boundary, which correlates with their preferred division axis, and cells at the periphery of the wing pouch tended to be elongated orthogonal to the proximo-distal axis, which also correlates with their preferred circumferential division axis (Fig 5C and 5D). Within the main part of the wing pouch, however, where mitotic spindles tend to align proximo-distally, pointing towards the dorsoventral boundary, no such elongation of the interphase cells was seen (Fig 5C and 5D). Thus there was no discernible correlation between cell division axis and cell shape within the wing pouch where our studies of the effects of cell death have been conducted. Similar results have been reported by others using different methods[10, 35].

**Fig 6:**
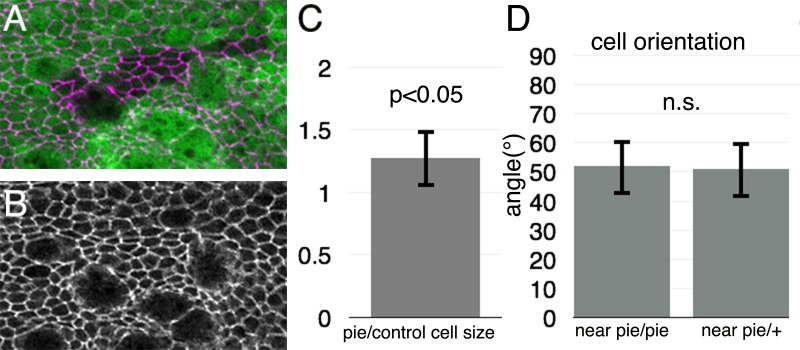
Effect of *pie* clones on cell size and orientation. **A**, *pie* homozygous clones lacking GFP. Cells outlined with anti-Dlg (magenta). **B,** Anti-Dlg labeling from panel A. The scattered cells with large apical profiles are mitotic cells, whose cell body rises apically to divide in the plane of the junctions. **C**, Apical area of *pie* homozygous cells compared to all other *pie*/+ and +/+ cells from similar proximo-distal locations in the wing pouch. On average, *pie/pie* cells were 1.27x larger. Error bar is 1 SEM. Significance assessed by paired sample t-test. **D,** Angle between clone boundaries and longest axes of cells surrounding clones. Cells adjacent to *pie*/*pie* clones were no more likely to be oriented towards the clones than cells surrounding control territories. Error bar is 1 SEM. Significance assessed by t test.

To examine whether cell death induces changes in cell shape within the wing pouch, we compared the size of cells within *pie* homozygous clones to those of *pie* heterozygous or wild type cells at comparable locations (Fig 6). A modest but statistically significant increase in the apical surface area of *pie* homozygous cells was observed compared to controls (Fig 6A–6C). To assess cell shape in the surrounding cells whose division orientation has been found to be affected, we determined the long axis of cells adjacent to *pie* homozygous clones in the wing pouch and its angle with respect to the clone. No significant elongation towards the clones was observed (Fig 6D).

## Discussion

In this paper we made use of the observation that clones of imaginal disc cells mutant for *pie*, which exhibit an elevated rate of apoptosis, bias the cell division orientation of other cells nearby in a search for a signal responsible for cell division orientation[8]. It is hypothesized that dying *pie* cells may be the source of a polarizing signal that is detected by other cells, and the roles of candidate signals were evaluated by removing them genetically from *pie* mutant cells. It is further hypothesized that the result may also be relevant to the orientation of cell divisions in normal development.

Since cell division orientation requires the PCP receptor Fat, we tested whether its PCP ligand Dachsous was required, but excluded this model. Since cell division orientation also requires Dachsous in the dividing cells, we also tested whether Fat itself was a signal required in the apoptotic clones but excluded this also. In fact both Fat and Dacshous could be eliminated together from the dying cell population without preventing the orientation of nearby cells (Fig 2). We next considered the possibility that rather than expressing Fat or Dachsous, apoptotic cells might down-regulate one or both proteins and that this might affect nearby cells, but found that eliminating one or both genes was not sufficient to orient nearby cell divisions (Fig 3). We did not test the possible contribution of Four-jointed, a kinase that phosphorylates Fat and Dachsous proteins in the Golgi, because Four-jointed should be unable to signal in cells already mutated for both *ft* and *ds* [54, 55]. Altogether, the experiments eliminated known ligands for the Fat/Dachsous PCP pathway, suggesting that the pathway must be required to orient cell division in response to some other signal.

It has been suggested that apoptotic imaginal disc cells secrete the morphogens Dpp and Wg in the process of stimulating compensatory proliferation[41, 48, 49]. Since Dpp and Wg pattern many aspects of imaginal disc development, including the expression of some PCP genes [50], they were candidates to orient the division of imaginal disc cells. Contrary to this prediction, clones of apoptotic cells lacking Dpp and Wg continued to orient nearby cell divisions (Fig 4). We cannot exclude that there may be other biochemical signals from dying cells that orient cell division. For example, there are other Wnt proteins in *Drosophila* that might affect cell polarity[56].

One other model consistent with these results is that cell division is oriented by physical constraints rather than biochemical signals[10, 35]. It is thought that in the wild type wing disc, the characteristic circumferential division pattern of the peripheral cells is a result of their being stretched around the growing wing pouch[10, 35]. Consistent with this conclusion, it has been reported that when a clone of cells grows more rapidly than the surrounding epithelium, cells around the clone are stretched circumferentially to accommodate the hyperplastic region, and this change in shape tends to orient cell divisions in a circumferential pattern around the hyperplastic clones[10]. By analogy to these findings concerning enhanced growth, it might be expected that clones of cells experiencing high rates of cell death would expand more slowly than surrounding cells, and that this would stretch the cells around the clone inwards towards the slow growing region, leading to a reorientation of cell divisions towards the slow growing clone, opposite to the case of more rapidly growing clones. As expected given their persistent cell death, clones of *pie* homozygous cells grow more slowly than control clones, and exhibit a small increase in apical cell size, consistent with local tension in the epithelium (Fig 6A–6C). We have reported the changed orientation of cell division near to pie clones previously[8]. We were unable here, however, to measure a consistent change in shape of the wing cells adjacent to *pie* homozygous clones, the population of cells where the altered division orientation is measured (Fig 6D). This lack of correlation between cell shape and cell division orientation is also seen for wing pouch cells in the wild type wing disc, which show a proximo-distal division preference but no obvious proximo-distal polarization[35, 36]. We did not measure the shapes of mitotic cells separately, and so cannot exclude the possibility that only the mitotic cells exhibit altered shapes in the wing pouch[36]. Recently, it has been reported that the orientation of epithelial cell division is determined by microtubule interactions with cell junction vertices, and that cell shape is a poor predictor of cell division in rounded cells, where the disposition of cell junction vertices varies[9]. This may explain why both the normal cell division orientation and the response to cell death do not correlate with cell shape within the wing pouch region, where cells are more rounded than in peripheral regions of the wing disc.

Oriented cell divisions are suggested to contribute to organogenesis[4, 57]. It was suggested that oriented cell divisions are responsible for the shape of cell clones in the wing disc, which ultimately determines the shape of the whole tissue (which is a collection of clones)[4]. Oriented cell divisions may have other functions, for example they may represent a homeostatic mechanism that ameliorates growth-induced mechanical stress[5–7].

The shape of cell clones becomes less regular during cell competition, and the interfaces between wild type and Minute cell populations become more convoluted and interdigitated [8, 30]. Previously, we suggested that oriented cell division could be responsible for the intermingling of wild type and Minute cells[8]. Recently, Levayer et al described very similar intermingling between cells in the pupal notum that are induced to compete by expression of different levels of Myc protein[31]. Very little cell division occurs in pupal notum, and Levayer et al describe cell neighbor exchanges that are responsible for intermingling the cell populations. They propose these exchanges are promoted by mechanical effects of differential growth rates[31]. Wild type and Minute cells also grow at different rates[25], but the apoptotic protein baculovirus p35 reduces the degree of intermixing between wild type and Minute cells[8]. There is now evidence that p35 also stimulates Minute growth rate, while having less effect on wild type cells[58]. Although the precise mechanism is unclear, Minute cell growth is possibly stimulated by signals from the undead *Rp/Rp* cells that are preserved when p35 is expressed[59]. Together these data raise the possibility that p35 may affect both cell division orientation and intermingling of wild type and Minute cells by equalizing their relative growth rates. In the case of *pie* clones that expand slowly, differential growth might result in local mechanical stretching which influences nearby cell divisions, although it can’t be excluded that the *pie* mutant clones have other differences from wild type.

Fat has a role as an upstream regulator of the Salvador-Warts-Hippo (SWH) pathway of tumor suppressors[18, 60–63]. There is substantial evidence that the SWH pathway responds to mechanical cues. Inputs are reported from actin polymerization status and from adhesion junctions via alpha-catenin and Juba proteins[64, 65]. Recent studies indicate that the SWH pathway itself promotes epithelial junctional tension, which is reduced in clones of *ft* or *wts* mutants[66]. Cell division orientation also depends on *atro*, however, which has been thought not to affect SWH activity, since it does not affect growth[8]. Recent studies suggest that mutations in the Fat-Dachsous pathway may affect PCP through a disruption of the Spiny Leg protein by de-repressed Dachs that is not a reflection of normal Dachs function[67, 68]. This does not explain how cell division orientation is affected by Fat or Dachsous but it does raise the possibility that Fat and Dachsous mutations might affect processes that depend little on their normal alleles. What we report here is that the model developed for planar cell polarity, in which ligand-receptor interactions between Fat and gradients of Dachsous control cell polarity, do not seem applicable to the orientation of cell division in the wing disc, where mechanical factors may be important.
